# Structural basis of arrestin-3 activation and signaling

**DOI:** 10.1038/s41467-017-01218-8

**Published:** 2017-11-10

**Authors:** Qiuyan Chen, Nicole A. Perry, Sergey A. Vishnivetskiy, Sandra Berndt, Nathaniel C. Gilbert, Ya Zhuo, Prashant K. Singh, Jonas Tholen, Melanie D. Ohi, Eugenia V. Gurevich, Chad A. Brautigam, Candice S. Klug, Vsevolod V. Gurevich, T. M. Iverson

**Affiliations:** 1 Department of Pharmacology, Vanderbilt University, Nashville, TN 37232 USA; 2 Department of Biophysics, Medical College of Wisconsin, Milwaukee, WI 53226 USA; 3 Department of Cell and Developmental Biology, Vanderbilt University, Nashville, TN 37232 USA; 4 University of Applied Sciences Emden/Leer, Emden, 26723 Germany; 5 Department of Biochemistry, Vanderbilt University, Nashville, TN 37232 USA; 6 Center for Structural Biology, Vanderbilt University, Nashville, TN 37232 USA; 7 Departments of Biophysics and Microbiology, The University of Texas Southwestern Medical Center, Dallas, TX 75390 USA; 8 Vanderbilt Institute of Chemical Biology, Vanderbilt University, Nashville, TN 37232 USA; 9 Present Address: Louisiana State University, Baton Rouge, LA 70803 USA

## Abstract

A unique aspect of arrestin-3 is its ability to support both receptor-dependent and receptor-independent signaling. Here, we show that inositol hexakisphosphate (IP_6_) is a non-receptor activator of arrestin-3 and report the structure of IP_6_-activated arrestin-3 at 2.4-Å resolution. IP_6_-activated arrestin-3 exhibits an inter-domain twist and a displaced C-tail, hallmarks of active arrestin. IP_6_ binds to the arrestin phosphate sensor, and is stabilized by trimerization. Analysis of the trimerization surface, which is also the receptor-binding surface, suggests a feature called the finger loop as a key region of the activation sensor. We show that finger loop helicity and flexibility may underlie coupling to hundreds of diverse receptors and also promote arrestin-3 activation by IP_6_. Importantly, we show that effector-binding sites on arrestins have distinct conformations in the basal and activated states, acting as switch regions. These switch regions may work with the inter-domain twist to initiate and direct arrestin-mediated signaling.

## Abstract (web summary)

While arrestins are mainly associated with GPCR signaling, arrestin-3 can signal independently of receptor interaction. Here the authors present the structure of arrestin-3 bound to inositol hexakisphosphate (IP_6_) and propose a model for arrestin-3 activation.

## Introduction

Arrestins modulate G protein-coupled receptor (GPCR) signaling in two ways. First, arrestins bind to activated, phosphorylated receptor and sterically block G protein coupling, terminating G protein activation^1^. Second, receptor-bound non-visual arrestins (arrestin-2 and -3, a.k.a. β-arrestin-1 and -2) initiate G protein-independent signaling^2^ via >100 proteins^3^. This arrestin-mediated signaling regulates cell proliferation and apoptosis^2^.

GPCR-dependent arrestin signaling occurs in the context of a complex between arrestin and phosphorylated, activated GPCR. In classic biochemical work on rhodopsin and arrestin-1, this arrestin–receptor interaction was found to rely on arrestin “sensors” that detect the phosphorylation and activation of the receptors. The phosphate sensor is formed by a group of positively charged side chains on the concave side of the N-domain that directly bind receptor-attached phosphates and is relatively unselective for the underlying sequence^4^. The activation sensor distinguishes between active and inactive GPCRs. To our knowledge, neither the identity of the activation sensor nor how it recognizes >800 distinct GPCRs have been proposed. These phosphate and activation sensors are believed to act synergistically, as the simultaneous triggering of both sensors elicits the highest-affinity receptor binding^5^.

Structural studies in the context of biochemical work suggest that triggering the phosphate and activation sensors of arrestin promotes a conformational change. Structures of all four vertebrate arrestins in the basal state^4, 6–8^ showed that arrestin is a two-domain protein with limited interaction between domains. Two inter-domain interactions have been suggested as key for stabilizing the basal conformation. The first is a group of buried charged side chains known as the “polar core”^7^. The second is a sequence in the C-terminus of arrestin termed the “C-tail” (residues 385–393 of arrestin-3) that binds to the N-domain in a way that both contributes a charge to the polar core and blocks access to the phosphate sensor^7^. Perturbation of either the polar core or the C-tail shifts the arrestin equilibrium to favor activation, suggesting that the inter-domain arrangement changes during activation^9^.

Recently, several strategies to stabilize active arrestin allowed structural characterization. These included the use of: (1) a more easily activated (termed “pre-activated”) splice variant of visual arrestin-1 called p44^10^, which is truncated before the C-tail; (2) an antibody to stabilize arrestin-2 bound to a vasopressin receptor phosphopeptide^11^; (3) the R175E mutant of arrestin-1 which has a destabilized polar core^12^; and (4) arrestin-1 with activating mutations tethered to constitutively active rhodopsin^13^. In the structures of p44, the phosphopeptide-bound arrestin-2, and the receptor-bound arrestin-1, a ~ 20° inter-domain rotation was observed, identifying inter-domain rotation as a hallmark of arrestin activation^10, 11, 13^. In the R175E variant, a ~ 7.5° inter-domain rotation suggests that this structure represents an activation intermediate^12^.

Despite these advances, many fundamental questions in arrestin-mediated signaling remain unanswered. Non-visual arrestins bind >800 distinct GPCRs, yet it is not clear how this broad receptor specificity is achieved. It is also unclear how the phosphate and activation sensors elicit the inter-domain rotation that accompanies arrestin activation, or how the active arrestin conformation initiates signaling. Finally, the arrestin-3 isoform is uniquely able to signal independently of GPCRs^14–18^. Previous studies identified that receptor-independent arrestin-3 activation biases signaling toward the activation of c-Jun N-terminal Kinase-3 (JNK3)^14, 16^. However it is neither clear how arrestin-3 is activated in the absence of GPCRs nor are there compelling hypotheses for why receptor-independent arrestin-3 activation preferentially initiates the JNK3 signaling cascade. Indeed, it is not clear how arrestin activated by any input correctly directs signaling toward one out of >100 downstream effectors.

To address these questions, we determined the 2.4 Å resolution crystal structure of arrestin-3 in complex with the non-receptor activator inositol hexakisphosphate (IP_6_). We demonstrate that the same sensors critical for detecting the phosphorylated and activated state of GPCRs are triggered during receptor-independent activation, and reveal how the triggering of these sensors elicits inter-domain rearrangements. Moreover, we identify properties of the arrestin activation sensor that allow broad specificity for GPCRs. Finally, we propose a mechanism for arrestin-dependent signal initiation and bias. Collectively, these findings address many outstanding questions in the arrestin field.

## Results

### Structure of IP_6_-activated arrestin-3

**Fig. 1:**
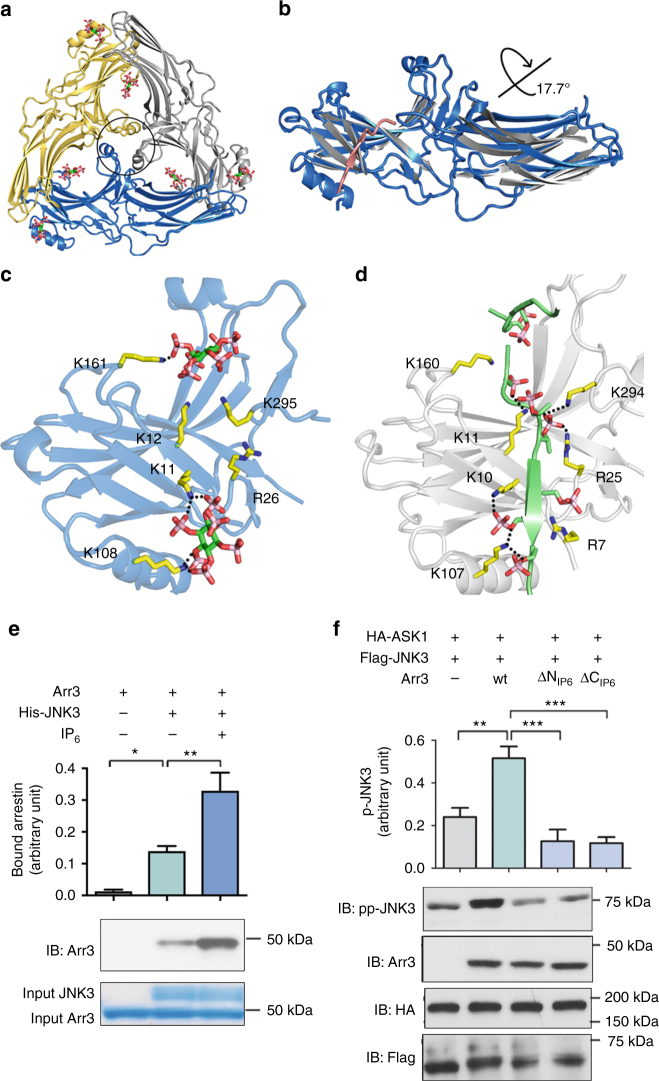
The structure of IP_6_-activated arrestin-3. **a** Ribbon diagram of the IP_6_-activated arrestin-3 (Arr3) trimer. Each protomer is shown in a different color (gray, yellow, blue); IP_6_ is shown as sticks. Finger loops are circled. **b** Overlay of the N-domain of IP_6_-activated (blue) with basal arrestin-3 (gray with a pink C-tail, PDB 3P2D^6^) highlights the 17.7° inter-domain rotation. **c** IP_6_ binding sites within the phosphate sensor on the arrestin-3 N-domain. **d** Phospho-peptide binding sites within the phosphate sensor on arrestin-2 N-domain (PDB entry 4JQI^11^). **e** Arrestin-3 binding to purified His_6_-JNK3 immobilized on Ni-NTA resin in the presence or absence of 100 µM IP_6_ (*n* = 8). The quantity of bound arrestin-3 was measured by densitometry (mean ± SEM) and compared using one-way ANOVA. *p ≤ 0.05; **p ≤ 0.01. **f** Measurement of JNK3 activation by arrestin-3 and mutants of the IP_6_ binding sites. JNK3 activation (mean ± SEM) was assessed by measuring the phospho-JNK3 (ppJNK3) level in HEK293 cells co-transfected with HA-ASK1, Flag-JNK3 and either empty vector, arrestin-3, the ΔN_IP6_ arrestin-3 (R8Q, K11A, K12A, K108Q, K161Q, K295Q) or the ΔC_IP6_ arrestin-3 (K233Q, R237Q, K251Q, K325Q, K327Q). The assay was repeated three times and JNK3 phosphorylation was compared using one-way ANOVA. **p ≤ 0.01, ***p ≤ 0.001

GPCR-independent arrestin-3 initiation of the JNK3 cascade is established in the literature^14–18^. Although a physiological non-receptor activator of arrestin-3 has not been unambiguously identified, inositol phosphates and heparin have been suggested as possibilities^19^. To test whether IP_6_ could activate arrestin-3, we determined the structure to 2.4 Å resolution (Fig. 1a; Table 1), which shows that the IP_6_-arrestin-3 complex is a trimer (Fig. 1a). In the structure, we identified electron density consistent with two bound IP_6_ molecules per arrestin (Supplementary Fig. 1a, b); each IP_6_ molecule is located between the protomers of the arrestin trimer (Fig. 1a). The structure shows that IP_6_ binds to arrestin-3 at the same location as C-tail in basal arrestin such that IP_6_ displaces the C-tail (Supplementary Fig. 1c). The IP_6_-bound structure exhibits an inter-domain twist of 17.7° with respect to the basal conformation (Fig. 1b). Moreover, the IP_6_ binding sites in the N-domain overlap with the binding sites for phosphorylated receptor (Fig. 1c, d). These observations are consistent with this structure representing activated arrestin-3.

**Table 1:** Summary of crystallographic data collection and refinement statistics^a^

*Data collection*	
Beamline	LS-CAT ID-D
Wavelength	1.7394 Å
Resolution (Å)	50.0–2.4
Space group	P6_3_
Unit cell dimensions	*a* = *b* = 97.575 Å, *c* = 76.938 Å
R_sym_	0.051 (0.546)
R_pim_	0.031 (0.416)
CC_1/2_^b^	0.865
I/σ	26.2 (2.00)
Completeness	97.5 (96.0)
Redundancy	3.4 (2.6)
*Refinement*	
R_cryst_^c^	0.210
R_free_	0.243
RMS deviation from ideal	
Bond lengths	0.004 Å
Bond angles	1.09°
Ramachandran statistics	
Most favored	95.0%
Additionally allowed	4.5%
Disallowed	0.6%

^a^Numbers in parenthesis are values for data in the outer resolution shell, corresponding to 2.44–2.40 Å resolution

^b^CC_1/2_ is only reported for the outer resolution shell

^c^R_cryst_ = Σ||F_obs_| − |F_calc_||/ΣF_obs_. R_free_ is the same as R_cryst_ for a set of data omitted from the refinement

### IP_6_ binding supports GPCR-independent activation in cells

As IP_6_-bound arrestin-3 adopts an active conformation, we tested whether IP_6_ binding enhances effector binding in vitro and induces arrestin-3-dependent JNK3 activation^14–18^ in cells. To identify whether IP_6_ enhances JNK3 binding, we compared the capacity of column-immobilized His_6_-JNK3 to bind arrestin-3 in the presence or absence of IP_6_ (Fig. 1e). Even in the absence of IP_6_, arrestin-3 binds to JNK3, as described in^17, 20^. This may reflect either low-affinity binding of JNK3 to the basal arrestin-3, or the ability of arrestin-3 to spontaneously sample the active conformation in the absence of activator. We observed an approximate doubling of the arrestin-3 binding to JNK3 in the presence of IP_6_ (Fig. 1e). This is consistent with IP_6_ shifting the conformational equilibrium of arrestin-3 to an active state that binds JNK3.

We next mutagenized the IP_6_ binding residues in the N-domain (ΔN_IP6_; Fig. 1c) or the C-domain (ΔC_IP6_, Supplementary Fig. 1d), then tested the impact on receptor-independent JNK3 activation in HEK293 cells (Fig. 1f). Co-expression of wild-type arrestin-3 with ASK1 (an upstream kinase in the JNK3 cascade^21^) and JNK3 resulted in robust JNK3 phosphorylation. This was significantly attenuated in cells expressing the ΔN_IP6_ or ΔC_IP6_ arrestin-3 variants, providing strong evidence that IP_6_ activates arrestin-3 in cells, and facilitates arrestin-dependent JNK3 activation.

To rule out alternative interpretations for the loss of function in the arrestin-3 variants, we performed two types of assays. First, we tested whether the affinity of arrestin-3 for IP_6_ would allow binding at physiological concentrations of IP_6_. Second, we ensured that the ΔN_IP6_ and ΔC_IP6_ variants specifically reduced receptor-independent signaling and had no global signaling defect.

To determine whether arrestin-3 binds IP_6_ at the concentrations found in cells, we used microscale thermophoresis (MST) to measure binding affinity. We labeled wild-type arrestin-3 with AlexaFluor C5 maleimide dye, titrated IP_6_ into fluorophore-labeled arrestin-3, activated with an infrared laser, and monitored the fluorescence intensity. MST allowed us to obtain binding curves and calculate equilibrium dissociation constants. Wild-type arrestin-3 exhibits two binding affinities for IP_6_ (K_D_ = 57 nM and 90 μM; Supplementary Fig. 1e). As intracellular IP_6_ concentrations range between 35 and 105 µM^22, 23^, these affinities are consistent with IP_6_ being able to occupy both binding sites in cells.

To ensure that that the mutants had no global signaling deficit, we evaluated folding and rhodopsin binding of purified wild-type and mutant arrestin-3 in vitro. As the IP_6_ binding sites in the arrestin-3 N-domain overlap with the binding sites for phosphorylated receptor (Fig. 1c, d), we anticipated that the ΔN_IP6_ variant would exhibit dramatically reduced receptor binding. However, the binding sites in the C-domain are unique to IP_6_, and if this variant is correctly folded and functional, the ΔC_IP6_ mutations should only have a minor impact on receptor association. As anticipated, the ΔC_IP6_ arrestin-3 variant retained significant receptor binding, while ΔN_IP6_ arrestin-3 mutant showed a substantial reduction (Supplementary Fig. 1f) but was properly folded. Collectively, these experiments support the importance of the IP_6_-binding sites in receptor-independent arrestin-3 signaling.

### IP_6_ triggers the arrestin-3 phosphate sensor

**Fig. 2:**
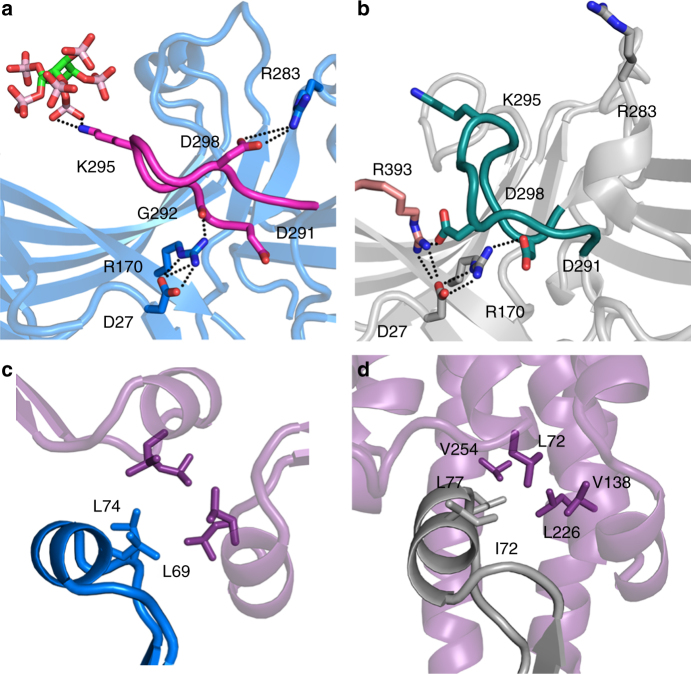
The phosphate and activation sensors in IP_6_-activated arrestin-3. **a** Conformation of the lariat loop in IP_6_-activated arrestin-3 disrupts the polar core. **b** Conformation of the lariat loop in basal arrestin-3 (PDB 3P2D^6^) with an intact polar core. **c** Helical conformation of the finger loop stabilized by the active arrestin-3 trimer. **d** Similar hydrophobic contacts are observed in the arrestin-1-rhodopsin interaction (4ZWJ^13^), and have distances of 3.5–4 Å between hydrophobic side chains

The IP_6_ molecules make extensive contacts with arginine and lysine side chains on the arrestin-3 N-domain (Fig. 1c). Critically, phosphate binding recruits Lys295 of the lariat loop, which contains two out of five charged residues in the polar core (Fig. 2a, b). This interaction likely elicits a conformational change that disrupts the polar core and triggers the phosphate sensor (Fig. 2a, b). A comparison of the IP_6_-bound structure to the reported structure of arrestin-2 with a vasopressin receptor phosphopeptide shows that the positions of the IP_6_ phosphates closely resemble those of the peptide-attached phosphates (Fig. 1c, d)^11^. The structure of arrestin-3 in the basal conformation^6^ shows that the C-tail binds to the N-domain, sterically occluding the phosphate binding sites (Supplementary Fig. 1c), but does not recruit Lys295. As a result, the polar core remains intact (Fig. 2b). Thus, IP_6_ and polyphosphated receptors bind to the same arrestin elements and trigger the phosphate sensor via the same mechanism by: (1) recruiting phosphate-binding side chains, (2) altering the conformation of the lariat loop, and (3) disrupting the polar core (Fig. 2a, b). This helps explain how relatively disparate phosphorylated species promote arrestin activation and helps clarify how the C-tail stabilizes the basal conformation.

Intriguingly, the Benovic and Brenner groups showed that soaking crystals of arrestin-2 with IP_6_ did not trigger the phosphate sensor^24^. Comparing the binding locations of the IP_6_ in arrestin-2 vs. arrestin-3^24^ shows that one of the IP_6_ molecules binds arrestin-2 in a different location (Fig. 1c, Supplementary Fig. 1d, g, h). As a result, IP_6_ binding to the arrestin-2 does not recruit Lys294 (equivalent to arrestin-3 Lys295). This leaves the polar core intact despite strict conservation of phosphate binding residues. While it is not clear how IP_6_ binding is directed differently in these isoforms, it is consistent with reports that arrestin-2 does not support receptor-independent signaling^15, 16, 21^.

### Engagement of the arrestin-3 activation sensor

We wanted to explore whether the activation sensor is also triggered in IP_6_-activated arrestin-3. The activation sensor of arrestins is proposed to distinguish between active and inactive GPCRs^5^, although its identity has not been suggested in the literature. To offer insight into the identity of the activation sensor, we analyzed differences between active and inactive receptors. The most striking structural difference is the accessibility of a hydrophobic pocket on the intracellular side of the protein unique to active GPCRs^25^. This pocket represents a major site of interaction with both G proteins^26, 27^ and arrestins^13, 28^. In fact, it contributes 596 Å^2^ out of a total of 1362 Å^2^ of buried surface area in the rhodopsin-arrestin complex^15^. Cocrystal structures of opsin with pre-activated arrestin-1 or an arrestin-derived peptide suggest that the hydrophobic pocket binds to an α-helical conformation of the finger loop^13, 28^. However, there is controversy on this because of the quality of the electron density for this helix in these structures (Supplementary Fig. 2a, b). The α-helix of the finger loop contains a ED(I/L)D motif (residues 68–71 of arrestin-3)^13, 28^ that lacks secondary structure in basal arrestins^4, 6–8^ and is not altered in partially activated arrestin^29^. Helix formation of the finger loop presents several hydrophobic residues to the intracellular hydrophobic pocket of activated receptor^13, 28^.

Similarly, in the IP_6_-mediated trimer the finger loop is presented as an α-helix (Fig. 2c, d). In contrast with the receptor-bound structure^13^, this helix is associated with clear electron density (Supplementary Fig. 2c). In this conformation, hydrophobic residues would be exposed to solvent if the IP_6_-activated arrestin-3 were a monomer, but in the context of the trimer, these form Van der Waals (<4 Å) self-contacts around the molecular three-fold axis (Fig. 2c) and are shielded from solvent. This suggests that receptor-independent trimerization and the GPCR hydrophobic pocket stabilize the triggered activation sensor in the same way (Fig. 2c, d).

### IP_6_-mediated arrestin-3 trimerization and activation

If trimerization stabilizes the triggered activation sensor, then the trimer should be quite stable in the presence of IP_6_. It would also be critical for receptor-independent activation of arrestin-3. Importantly, the IP_6_-bound arrestin-3 trimer buries 3053 Å^2^ of surface area per protomer, a value indicative of a biologically relevant oligomer^30^. The trimer does not appear to be influenced by crystal contacts (Supplementary Fig. 3a); however, it does require the inter-domain twist of active arrestin. Indeed, modeling the basal conformation causes the misalignment of the IP_6_ binding sites (Supplementary Fig. 3b). This would be predicted to reduce IP_6_ affinity and therefore disfavor trimerization. We therefore next explored the stability of the arrestin-3 trimer in solution.

**Fig. 3:**
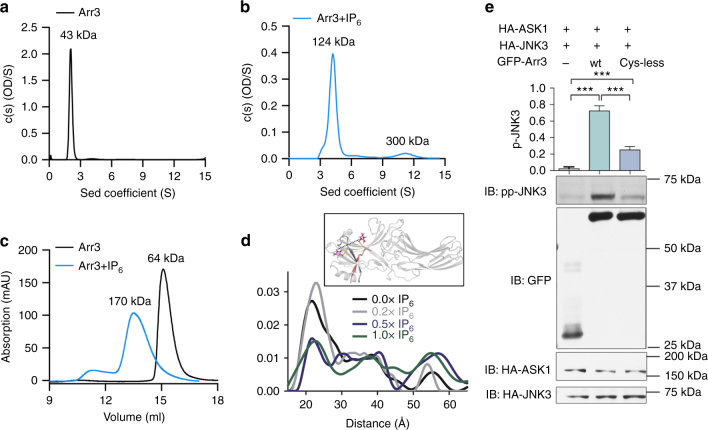
IP_6_ mediated trimerization and receptor-independent activation in cells. **a**-**b** Sedimentation velocity analytical ultracentrifugation (SVAUC) of arrestin-3. Measurements were performed in triplicate. **a** Representative SVAUC run in the absence of IP_6_ predicts a molecular weight matching a monomer. **b** In the presence of 100 µM IP_6_, the predicted molecular weight is consistent with a trimer. **c** Representative size exclusion chromatograms of arrestin-3 (Arr3) in the presence and absence of IP_6_. In the absence of IP_6_, 60 µM arrestin-3 (black) elutes at volume corresponding to the Stokes radius of a globular protein with a molecular mass of 64 ± 5.9 kDa. In the presence of 100 μM IP_6_, the elution volume is consistent with a globular protein of molecular mass 169.6 ± 9.7 kDa. The ratio of these molecular weights is consistent with IP_6_-dependent trimer formation. Measurements were performed in triplicate, errors are ± SEM. **d** Plot of the probability of the distances between spin labels at S13 and A392 for 100 µM arrestin-3 in the presence of the indicated molar ratios of IP_6_; inset shows the location of the spin labeled sites in basal arrestin-3 (PDB entry 3P2D^6^). **e** Comparison of JNK3 activation by GFP-arrestin-3 and Cys-less mutant. GFP-tagged arrestin allowed comparison of the expression levels of wild-type and variant arrestin-3, as described^14, 33^. JNK3 activation (mean ± SEM) was assessed by measuring the pp-JNK3 levels in COS7 cells co-transfected with HA-ASK1, HA-JNK3 and GFP or Venus-tagged wild-type and Cys-less arrestin-3. Assay was repeated five times and JNK3 phosphorylation was compared by one-way ANOVA followed by Bonferroni post hoc test with correction for multiple comparisons. ***p < 0.001

Using analytical ultracentrifugation, we found that the addition of IP_6_ to purified arrestin-3 converts the observed molar mass from 43 ± 1 kD (monomer) to 134 ± 5 kD (trimer) (Fig. 3a, b). Size exclusion chromatography (Fig. 3c, Supplementary Table 1) is also consistent with IP_6_-dependent trimerization of arrestin-3. We then explored the effect of arrestin-3 concentration on IP_6_-dependent trimerization and found that the trimer is stable at the lowest arrestin-3 concentration detectable by our instruments (~1 µM; Supplementary Fig. 3c).

We next assessed whether trimerization contributes to receptor-independent activation in cells using a trimerization deficient arrestin-3 variant. To design a trimerization deficient variant, we identified residues that are surface exposed in basal (monomeric) arrestin-3, buried in the trimer interface, and not associated with known biochemical functions of arrestin. This suggested Cys17 as a candidate side chain for targeting. We used a fully Cys-less variant of arrestin-3 because it has previously been shown to bind receptors normally^31^. This indicates that the Cys-less variant is folded and rules out many alternative interpretations of any results.

We first assessed whether the Cys-less variant lost the ability to form trimers, using size exclusion chromatography. We found that the Cys-less variant does not form stable trimers in response to IP_6_, but appears to shift to a molecular weight consistent with a dimer (Supplementary Fig. 3d). Comparing the arrestin-3 trimer to the arrestin-2 oligomers suggests that while the trimer can stabilize the inter-domain twist (Fig. 1a, b), other arrestin oligomers cannot. This makes Cys-less arrestin-3 suitable for measuring how trimerization impacts receptor-independent signaling.

To quantify IP_6_-dependent activation of Cys-less arrestin-3 in vitro, we employed double electron electron resonance (DEER) spectroscopy. One of the hallmarks of arrestin activation is the displacement of the C-tail from the N-domain upon the binding of phosphates to the phosphate sensor. We monitored this process via attached spin labels to cysteines replacing Ser13 in the N-domain and Ala392 in the C-tail. At these positions, the spin labels are separated by 22 Å in basal arrestin-3, when the C-tail is bound to the N-domain. Upon arrestin activation and C-tail release, these convert to a wide distribution of longer inter-spin distances. After titration with IP_6_, we found that only ~ 30% of the 22 Å distance converted to longer distances (Fig. 3d, Supplementary Fig. 3e), a ~ 70% loss in IP_6_-dependent activation. Cys-less arrestin-3 was reported to exhibit nearly 100% release of the C-tail in response to phosphorhodopsin in the same assay^31^, which rules out the possibility that Cys-less arrestin-3 is signaling deficient.

We then compared the ability of wild-type and Cys-less arrestin-3 to mediate receptor-independent JNK3 activation in cells. We observed a 65% reduction in JNK3 activation in cells expressing the Cys-less mutant as compared to wild-type arrestin-3 (Fig. 3e). This correlates with the loss of IP_6_-dependent activation measured by DEER.

Our observed trimer contrasts with reported multi angle laser light scattering (MALLS) of IP_6_-bound arrestin-3, which were explained by a dimer^32^. To investigate this discrepancy, we evaluated differences in the experimental design and identified that a protease inhibitor, benzamidine, used in the MALLS studies induces heterogeneous oligomerization (Supplementary Fig. 4a–c, details in the legend), with oligomers having an average molecular weight consistent with a dimer. As benzamidine is not present in cells, it is likely that these oligomers are non-physiological.

### The phosphate and activation sensors are intimately linked

With the phosphates of IP_6_ apparently triggering the phosphate sensor and trimerization likely triggering the activation sensor, we wanted to explore the relationship between IP_6_-binding and trimerization. Since IP_6_ molecules mediate trimer formation, we propose that IP_6_-binding and trimerization are intertwined. This would suggest that triggering the phosphate and activation sensors are linked during receptor-independent arrestin-3 activation.

**Fig. 4:**
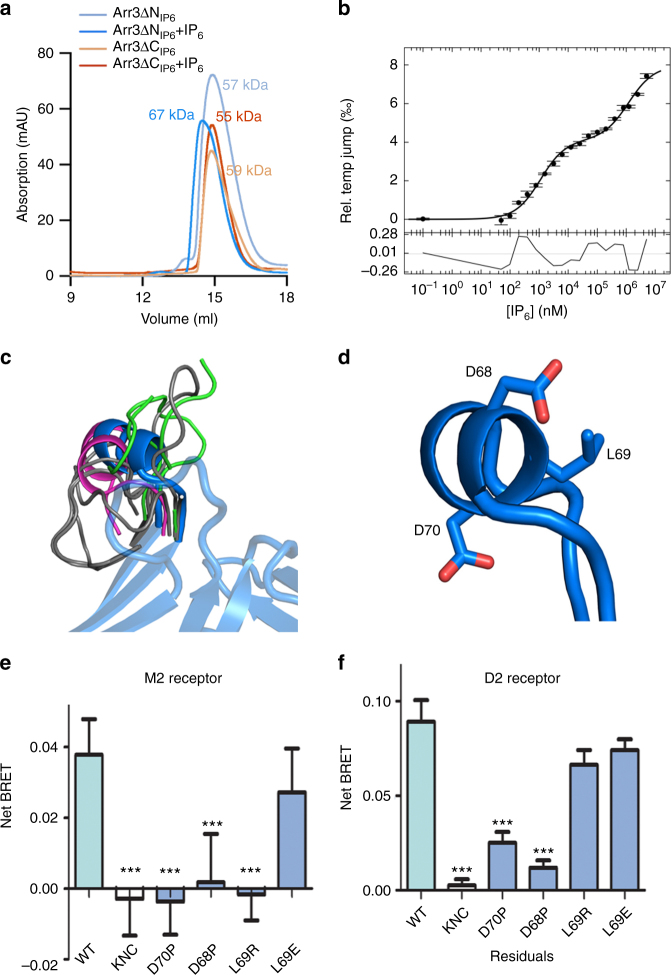
The interplay between phosphate and activation sensors in receptor-independent and receptor-dependent signaling. **a** Size exclusion chromatography (SEC) of the ΔN_IP6_ and ΔC_IP6_ mutants measured on a Superdex S200 Increase 10/300 GL column (24 mL). Arrestin-3 (1–393) runs anomalously on size exclusion chromatography, but exhibits a characteristic shift in molecular weight upon the addition of IP_6_. In the absence of IP_6_, both the ΔN_IP6_ and the ΔC_IP6_ mutants are monodisperse and have a similar elution volume to wild-type, but in the presence of IP_6_, no mobility shift is observed. **b** Temperature-jump binding curve for the Cys-less-T222C arrestin-3. **c** Overlay of finger loop of arrestin structures. Basal (gray): PDB entries 1CF1^7^, 1JSY^35^, 1ZSH^24^, 3P2D^6^, 1G4M^4^; active (green): PDB entries 4ZRG^12^, 4JQI^11^, 4J2Q^10^. Bound to receptor (magenta): 4ZWJ^13^. Bound to IP_6_ (blue) **d** The conserved motif EDL/(I)D folds into an α-helix. **e,f** Evaluation of mean binding ± SEM of wild-type and mutant Venus-arrestin-3 binding to the luciferase-tagged **e** M2 muscarinic or **f** D2 dopamine receptor by BRET. In arrestin-3-KNC (K11A, K12A, L49A, D51A, R52A, L69A, Y239A, D241A, C252A, P253A, D260A and Q262A), two key phosphate-binding lysines and 10 residues that bind other parts of the receptor were mutated to alanines. This precludes GPCR binding as described^22, 34^, making this an appropriate negative control. Data from three experiments were compared to wild-type by one-way ANOVA. ***p ≤ 0.001

To test this, we first evaluated the ability of the ΔN_IP6_ and ΔC_IP6_ variants of arrestin-3 to form IP_6_-dependent trimers. As described above, the IP_6_-binding sites are altered by mutagenesis in these variants, and receptor-independent JNK3 activation in cells is compromised (Fig. 1f). Size exclusion chromatography showed that these variants no longer trimerize in the presence of IP_6_, instead exhibiting mobility consistent with a monomer (Fig. 4a, Supplementary Table 1).

We next tested how loss of trimerization impacts IP_6_ affinity using MST. The Cys-less variant is a valuable tool for this measurement because trimerization is disrupted; however, our experimental design used a maleamide-conjugated fluorophore, which interacts with cysteines. Thus, we re-introduced a cysteine residue to allow labeling. The T222C variant was considered suitable for this purpose because T222 is distal from all characterized functional regions. This mutant is also monodisperse, consistent with correct folding (Supplementary Fig. 3c). When compared to wild-type arrestin-3 (K_D_ = 57 nM and 90 μM; Supplementary Fig. 1e), the T222C-Cys-less variant shows substantially reduced IP_6_ binding affinity (K_D_ = 1.1 µM and 1.2 mM, Fig. 4b), consistent with trimerization contributing to IP_6_ affinity.

Collectively, these data strongly suggest IP_6_-binding and trimerization work together to support receptor-independent arrestin-3 activation of JNK3. Because IP_6_ appears to directly bind the phosphate sensor, and trimerization appears to stabilize a triggered activation sensor, we conclude that during receptor-independent activation, triggering of these sensors is intimately linked. This mirrors the observed synergy of these two sensors in receptor-dependent signaling^5^.

### Arrestin-3 sensors in broad receptor selectivity

There are >800 GPCRs, but only two non-visual arrestins (arrestin-2 and arrestin-3) that recognize the phosphorylated and activated forms of these receptors; arrestin-3 also recognizes non-receptor activators. Intuitively, the mechanism of broad receptor specificity of the phosphate sensor is straightforward, as the N-domain of arrestin can bind receptor-attached phosphates with the correct spacing. In contrast, it is less clear how the activation sensor might recognize this large number of receptors.

If our assignment of the finger loop as a part of the activation sensor is correct, this element must interact with the hydrophobic pocket on >800 GPCRs with limited sequence similarity. We therefore analyzed the finger loop to identify properties that could contribute to broad receptor recognition. An overlay of available arrestin structures suggests that the finger loop is on a flexible tether (Fig. 4c) and can be presented at many angles to an interaction partner. This could allow the finger loop to adapt to the different hydrophobic pockets on receptors. If so, the helicity, hydrophobicity, and flexibility of the finger loop would be predicted to contribute to the broad receptor specificity of the activation sensor. Hence, perturbation of these properties would reduce activation sensor-dependent receptor binding. We therefore designed mutations (Fig. 4d) that introduced (a) a helix-breaking proline (D68P), (b) a flexibility-reducing proline terminating the helix (D70P), or (c) a charge within the hydrophobic region (L69E/R). Modeling based on available structures^13, 28^ suggests that neither introduced proline should directly interact with the receptor.

We measured the binding of these variants to M2 muscarinic and D2 dopamine receptors, which we selected because they are more dependent upon the activation sensor than on receptor-attached phosphates for arrestin binding^33^. We used a bioluminescence resonance energy transfer (BRET) assay in COS7 cells cotransfected with luciferase-tagged receptors and Venus-tagged arrestin-3^14^ (Fig. 4e, f, Supplementary Fig. 5a, b). Wild-type arrestin-3 shows a robust increase in BRET signal upon agonist stimulation, whereas arrestin-3-KNC (a negative control with 12 substitutions of key receptor-binding residues that binds poorly to receptors^14^) does not^33, 34^. Mutants designed to perturb helix formation or flexibility had substantially reduced binding to both receptors (Fig. 4e, f). Interestingly, charged residues had different effects on binding to the M2 and D2 receptors. This variability may reflect the presence or absence of complementary charges in the inter-helical cavity of different GPCRs, or the strength of other interactions between receptor and arrestin-3.

As the finger loop is also a major contact for IP_6_-dependent arrestin-3 trimerization, we tested if finger loop variants affect receptor-independent activation and signaling. Alteration of the finger loop prevented IP_6_-dependent trimerization of the D68P variant, reduced trimerization of the L69R and D70P mutations, and had no detectable impact in the L69E variant (Supplementary Fig. 5c, d). JNK3 activation in cells was not affected in the D69R and D70P mutations (Supplementary Fig. 5e). One interpretation of this finding is that IP_6_-induced arrestin-3 activation is more dependent upon the phosphate sensor than the activation sensor, a property shared with many GPCRs^33^. However, given our data suggesting that triggering the phosphate and activation sensors during receptor-independent activation is closely linked, it is more likely that the finger loop of our variants is presented in a way that allows trimerization.

### Switch regions in arrestin-mediated signaling

A major functional consequence of non-visual arrestin activation is the engagement of downstream effectors, yet how activated arrestin supports signaling has never been explained. In the IP_6_-activated arrestin-3 structure we identified structural changes in known effector binding elements that may promote signal initiation. Activation-induced conformational changes in these regions have not been previously reported, although inspection of the coordinates for the active arrestin-1 and arrestin-2 indicates that similar changes accompany activation of other arrestins^10, 11, 13^. We suggest that these conformational changes act as molecular switches functionally analogous to the switch regions of G proteins [reviewed in 35], and therefore term these “arrestin switch” regions. In conjunction with the inter-domain twist, these conformational changes would create effector-binding sites and turn on signaling when arrestin is activated.

**Fig. 5:**
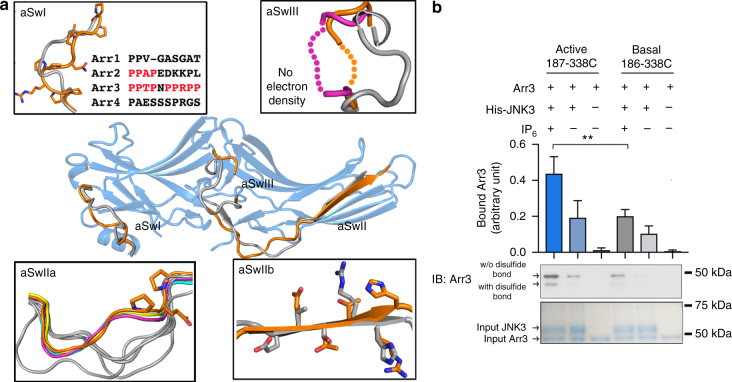
Arrestin switch regions. **a** Conformations of the arrestin switch regions in the active form (orange (IP_6_-activated arrestin-3 (Arr3)), magenta (phosphopeptide-activated arrestin-2; PDB 4JQI^11^), cyan (rhodopsin-activated arrestin-1; (PDB 4ZWJ^13^)), yellow (p44; PDB 4J2Q^10^)) differ from those in basal arrestin-3 (gray, PDB 3P2D^6^). Insets highlight switch regions. In the inset showing aSwI, the sequences in four bovine arrestin isoforms are shown. **b** Quantitation of arrestin-3 binding to His_6_-JNK3 in the presence and absence of IP_6_ (100 µM), monitored using an in vitro pull-down assay. JNK3 was immobilized on Ni^2+^ resin, exposed to arrestin-3 (with disulfides stabilizing active or basal aSwIIb), washed, then eluted in sample buffer and visualized by SDS-PAGE and Western. Disulfide trapped arrestin-3 (red box) was quantified separately by densitometry and the ratio was calculated (means ± SEM). **p ≤ 0.01. The overall amount was normalized by the ratio of disulfide trapped arrestin (Supplementary Fig. 5)

Arrestin switch I (aSwI; residues 89–97; Fig. 5a) is strongly conserved in arrestin-3 homologs from other species, but not in the other three arrestin isoforms (Fig. 5a, inset). In arrestin-3, this nine amino acid segment contains seven prolines, including two PPXP motifs that may be recognized by SH3 domains^35, 36^. Comparison of the basal^6^ and active conformations of aSwI reveals a maximal displacement of 5.8 Å and includes an unusual pair of tandem *cis* bonds (Pro94-Pro95 and Pro95-Arg96) in the best-fitting model. This rare structural feature is associated with conformational change^37, 38^. However, the electron density of this region is difficult to interpret, and not all residues could be modeled with confidence (Supplementary Fig. 6a). The flexibility of this switch may be important for adapting to different effectors.

Arrestin switch II (aSwII; residues 176–191; Fig. 5a) contains two distinct parts: IIa (residues 176–183) and IIb (184–191). ASwIIa connects the N- and C-domain, and has been proposed to act as a hinge between domains^39, 40^. ASwIIa also contains a polyproline motif (PQP residues 181–183) suggested as a non-canonical SH3 binding site in arrestin-2^35^. The aSwIIa shows a nearly identical conformation in all available active arrestin structures, but variable conformations in basal arrestins (Fig. 5a, inset).

ASwIIb includes the entirety of the first β-strand of the C-domain (residues 184–191) and superimposes in all active arrestin structures. As compared to most arrestins in the basal state, the β-strand of aSwII is register-shifted, which moves it one position in arrestin-2 and -3 and two positions in arrestin-1 (Supplementary Fig. 6b, c), although the active position of aSwIIb is observed in one crystal form of basal arrestin-2^24, 35^ (Supplementary Fig. 6b). Register-shifted β-strands are unusual, but have proposed roles in regulating signaling^41, 42^ and forming protein binding sites^43^.

Arrestin switch III (aSwIII, residues 307–316; Fig. 5a) is an extension of the lariat loop^7^, which is a part of the polar core that stabilizes the orientation of the two domains in the basal state. ASwIII becomes disordered in both activated arrestin-3 and arrestin-2^11^, a property commonly associated with the ability to mediate protein-protein interactions^44, 45^.

Using the same terminology, the arrestin C-tail may be considered aSwIV. In basal arrestins, the C-tail is anchored to the N-domain^4, 6–8, 35^. Receptor binding induces release of the C-tail, which becomes unstructured^31, 46, 47^. The binding sites for clathrin^48^ and clathrin adapter AP-2^48^ are localized in the C-tail of non-visual arrestins, and both become fully accessible upon C-tail release^49^.

To test the role of the switch regions, we focused on aSwIIb, and used disulfide trapping to stabilize the register-shifted β-strand (Supplementary Fig. 6d–f). We used the Cys-less arrestin-3 as background to avoid unintended disulfide bonds with native cysteines. We introduced a cysteine at position 338 and either position 186 (stabilizing the basal conformation) (Supplementary Fig. 6d), or position 187 (stabilizing the active conformation) (Supplementary Fig. 6e). Electrophoretic mobility suggests that disulfides formed at statistically identical levels in these variants (Supplementary Fig. 6f).

We then assessed the binding of each of these disulfide-stabilized versions of arrestin-3 to JNK3. In the presence of IP_6_, column-immobilized JNK3 exhibits increased binding to arrestin-3 with aSwII trapped in the active conformation (Fig. 5b). In the absence of IP_6_, the binding is lower for both variants, suggesting that the disulfide-trapped aSwII does not fully shift the arrestin equilibrium to an active state in the absence of IP_6_.

## Discussion

We used IP_6_-bound arrestin-3 to identify the structural requirements for receptor-independent arrestin activation, and to suggest how activation results in arrestin-mediated signaling. These data inform on several aspects of arrestin activation that have been enigmatic: how an arrestin can interact with many disparate receptor and non-receptor activators, adopt an active conformation, then initiate a variety of downstream signaling pathways.

Arrestin-3 is activated by >800 GPCRs plus non-receptor activators, including IP_6_. When coupling to receptors, arrestin acts as a coincidence detector that binds with high affinity to phosphorylated and activated receptors, as mediated by two independent sensors^5^. We show that the IP_6_ triggers the same two sensors. Indeed, the binding site for IP_6_ sterically overlaps with the binding site for receptor-attached phosphates (Fig. 1c, d). Similarly, the surface of the finger loop that interacts with the hydrophobic pocket of receptor also interacts with sister protomers in the trimer^13, 28^ (Fig. 2c, d). Because the same surfaces support receptor-independent trimerization and receptor binding, receptor-dependent and receptor-independent arrestin-3 activation appear to be mutually exclusive, i.e., the trimer does not bind receptors. The use of the same sensors and surfaces for trimerization and receptor binding also suggests that receptor-dependent and receptor-independent arrestin activation occur via a similar mechanism.

Structurally, arrestin activation involves several conformational rearrangements. First, negatively charged phosphates bind to the N-domain, displacing the C-tail and disrupting the polar core^11^. Our data and previous reports suggest the disruption of the polar core by phosphate binding is a hallmark of a triggered phosphate sensor^7, 50, 51^. Second, a hydrophobic environment induces the external presentation of hydrophobic residues of the finger loop on the central crest of the arrestin molecule, which folds into a short α-helix^13, 28^. Our data suggest that this helical finger loop is a hallmark of the triggered activation sensor. Finally, both receptor-dependent and receptor-independent arrestin activation induce an inter-domain twist in IP_6_-bound arrestin-3 (Fig. 1b) and all other activated arrestin structures^10–13^.

Molecular mechanisms connecting the phosphate and activation sensors to the domain twist have not been proposed. Our data suggest that each sensor acts via a distinct pathway. Triggering the phosphate sensor likely facilitates the inter-domain rotation in two ways. First, IP_6_ phosphates bind Lys295 on the lariat loop (Fig. 2a). This changes the loop position, removing two negative charges from the polar core (Fig. 2a, b). Second, IP_6_ directly displaces the C-tail, which contributes Arg393 to the polar core (Fig. 2b). Both changes break the polar core, making domain rotation possible.

**Fig. 6:**
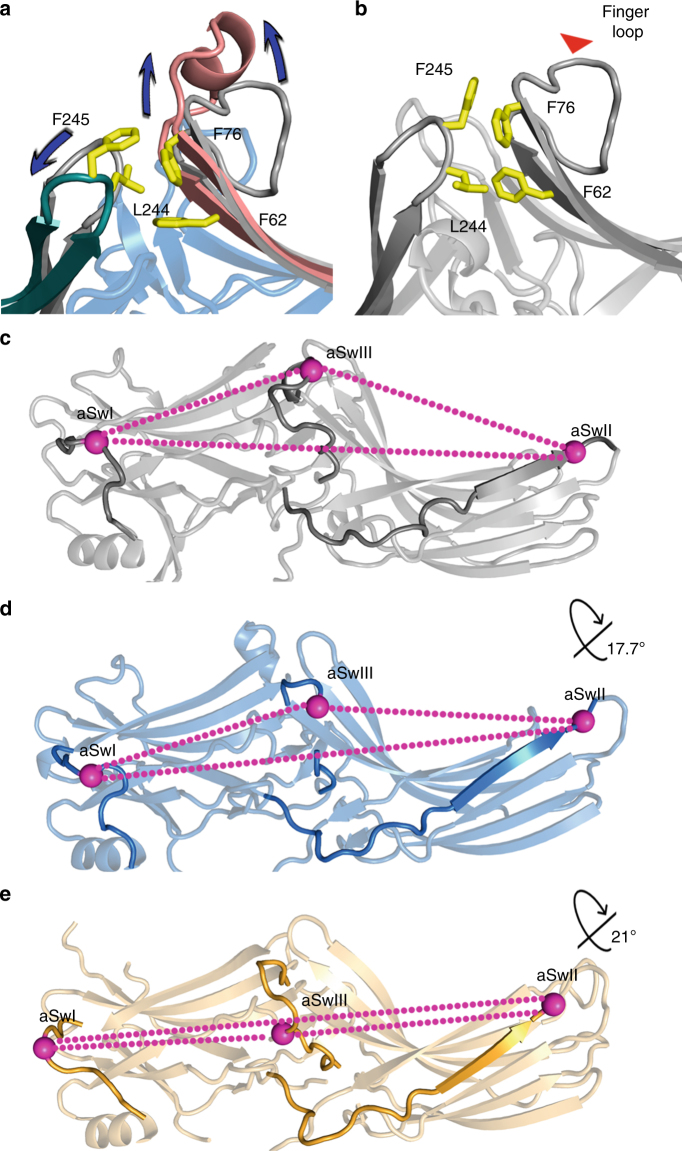
Mechanisms of arrestin activation and domain rotation. **a** Overlay of the hydrophobic core between β-strands supporting the finger loop (pink) and the C-loop (green) in the active arrestin-3 and in the basal arrestin-3 (gray, PDB 3P2D^6^). **b** Conformation of hydrophobic core between β-strands surrounding the finger loop and the C-loop in the basal arrestin-3 (PDB 3P2D^6^). **c**–**e** The inter-domain twist realigns the arrestin switch regions. In each panel, the switch regions are darker elements; magenta balls mark equivalent residues. **c** Basal arrestin-3 (PDB 3P2D^6^). **d** IP_6_-activated arrestin-3 with a 17.7° inter-domain rotation. **e** p44 arrestin-1 (PDB 4J2Q^10^) with a 21° inter-domain rotation

Triggering the activation sensor likely promotes inter-domain rotation by a complementary mechanism. An interaction between the finger loop and a hydrophobic environment can induce helix formation. The finger loop is part of the N-domain, but in basal arrestin, the hydrophobic residues are shielded from solvent via an interaction with a loop in the C-domain (residues 243–247, Fig. 6a, b). Activation-associated rearrangement of the finger loop alters this hydrophobic core, so that the β-strands surrounding the finger loop in the basal state shift in a manner promoting the domain rotation (Fig. 6a).

Based on this model, we propose how arrestins have achieved an inter-domain twist in reported structures (Supplementary Table 2). Our analysis suggests that in the IP_6_-bound arrestin-3, a physiologically relevant activator triggers both sensors, which appear to be highly interdependent. During receptor-induced activation, the sensors may act more independently, with some GPCRs relying more heavily on one sensor^33^. However, the two sensors likely act synergistically, as arrestins exhibit the highest binding when receptor is both activated and phosphorylated^5^.

The comparison of IP_6_-activated arrestin-3 and other active arrestin structures suggests a general model explaining how activated arrestins initiate and direct signaling. First, the inter-domain twist and the new positions of switches likely work in conjunction to form effector-binding sites. Because receptor-independent activation apparently biases signaling toward the JNK3 cascade^15–18, 21^, IP_6_-activated arrestin-3 is the first case where the signaling bias can be correlated with the structure. The inspection of other active arrestin structures suggests an intriguing possible mechanism of signal bias. The inter-domain twist is of different magnitude in available active structures, consistent with the proposed ability of active arrestin to adopt a range of conformations^9, 52, 53^. Moreover, conformations of the switch regions in our structure differ from those observed in other active arrestins. This ability to adopt a range of active conformations agrees with electron microscopy images of a chimeric β_2_-adrenergic receptor with arrestin-2^54^, fluorescence quenching binding studies of rhodopsin-arrestin-1^55^ and NMR spectroscopy of rhodopsin-arrestin-1^29^, all of which suggest heterogeneity of the receptor-arrestin complex.

Conceivably, signaling toward different effectors can be directed by combining distinct conformations of the arrestin switch regions with different inter-domain rotation angles (Fig. 6c–e, Supplementary Movies 1 and 2). The magnitude of each conformational change likely depends on the identity of the activator. In case of GPCRs, the conformations may be further influenced by the phosphorylation pattern of the receptor^52, 53, 56, 57^. Structures of arrestin in complex with a range of activators, including receptors with different phosphorylation patterns, are required to test this idea.

## Methods

### Materials

DNA modifying enzymes were from New England Biolabs (Ipswich, MA). DNA purification kits were from Zymo Research (Irvine, CA). HEK293 and COS7 cells are from ATCC. Cell culture reagents and media were from Mediatech (Manassas, VA) or Invitrogen (Carlsbad, CA). The luciferase substrate coelenterazine-*h* was from NanoLight (Pinetop, AZ). All other reagents were from Amresco (Solon, OH) or Sigma-Aldrich (St. Louis, MO). The primers used in this study are listed in Supplementary Table 3.

### Arrestin-3 expression and purification

Bovine arrestin-3 was cloned into the pTrcHisB vector (Invitrogen; Carlsbad, CA) with the codon for L394 mutagenized to a stop codon (TGA). This created a version of arrestin-3 truncated immediately after the C-tail attachment point (encoding residues 1–393 and deleting residues 394–408). This version of arrestin-3 has activation propensity indistinguishable from wild-type and was used to produce protein for the structure of the basal conformation of arrestin-3^6^. Because it has superior properties after purification, arrestin-3 (1–393) was used for the in vitro experiments in the manuscript, with the exception of a subset of the replicates that tested the effects of benzamidine. Only for these benzamidine experiments, one of the replicates arrestin-3 with a stop codon introduced at position R393. This variant of arrestin (1–392) is more easily activated. Experiments in cells used full-length arrestin-3 (1–408).

The pTrcHisB plasmid containing arrestin-3 (1–393) was transformed into E. coli BL21 Gold and grown in 1 L cultures in LB medium supplemented with 100 mg/L ampicillin. Cells were grown at 30 °C overnight with shaking at 250 r.p.m., then protein expression was induced with the addition of 35 µM IPTG for 4 h. Cells were collected by centrifugation and the pellet was stored at −80 °C.

Arrestin-3 was purified using a modification of a previously described protocol^58^. Briefly, cell pellets were resuspended in buffer containing 50 mM MOPS, pH 7.2, 5 mM EGTA, 2 mM tris-(2-carboxyethyl) phosphine (TCEP), and two protease inhibitor cocktail tablets (Sigma). Cells were disrupted by sonication at 4 °C. The lysate was clarified by centrifugation at 20,800×*g* (SLA 3000 rotor, RC-5B Plus centrifuge) for 60 min, and arrestin was precipitated by the addition of (NH_4_)_2_SO_4_ to a final concentration 0.32 mg/mL. Precipitated arrestin-3 was collected by centrifugation at 20,800×*g* (SLA 3000 rotor, RC-5B Plus centrifuge) for 90 min, and dissolved in buffer containing 10 mM MOPS pH 7.2, 2 mM EGTA, and 1 mM TCEP, then centrifuged again at 20,800×*g* (SLA 3000 rotor, RC-5B Plus centrifuge) for 60 min to remove particulates. The supernatant containing soluble arrestin-3 was applied onto a heparin column and eluted with a linear NaCl gradient. Fractions containing arrestin-3 were identified by SDS-PAGE and Western and combined. The salt concentration of the pooled fractions was adjusted to 100 mM, and the solution was loaded onto a linked HiTrap Q HP (GE healthcare) and HiTrap SP HP (GE healthcare) column. At a NaCl concentration of 100 mM, arrestin-3 flows through the Q column (while most contaminants bind), but binds the SP column. The columns were uncoupled and a linear NaCl gradient was used to elute arrestin-3 from the SP column. The fractions containing arrestin-3 were identified by SDS-PAGE and combined, concentrated with a 30 kDa cutoff concentrator, then further purified using a Superdex 200 increase 10/300 GL column (GE healthcare) equilibrated with 20 mM MOPS pH 7.2, 150 mM NaCl, and 2 mM TCEP. Folding and trimerization were monitored by size exclusion chromatography.

### Crystallography

Purified arrestin-3 (residues 1–393) was concentrated to 5 mg/mL and incubated with IP_6_ at 1:20 molar ratio for 30 min on ice. Crystals in the hexagonal space group P6_3_ were grown using the sitting drop vapor diffusion method by combining 2 µl of arrestin-3-IP_6_ and 2 µl of reservoir solution (100 mM Succinate/Phosphate/Glycine pH 8.5 and 25% PEG 1500). Microcrystals appeared within 24 h and were used for seeding. Crystals were harvested after 7 days, cryoprotected in 50% w/v glycerol and cryocooled by plunging into liquid nitrogen.

Diffraction data (Table 1) were collected at the Advanced Photon Source LS-CAT beamline 21-ID-D. Data were processed using HKL2000^59^. The structure was determined by molecular replacement in PHASER^60^ using isolated domains of a pruned version of arrestin-2 (PDB entry 1G4M^4^) as the search model. The best solution was associated with an initial R/R_free_ of 0.34/0.37 and was improved by model building in COOT^61^ and refinement in PHENIX^62^. The myoD conformation of IP_6_ was placed into difference electron density manually using the conformation found in PDB entry 4HNW. Figures were prepared in PYMOL.

### JNK3 activation measurements in HEK293 and COS7 cells

HA-tagged ASK1 and Flag-tagged JNK3 were co-transfected with either: (1) empty vector; (2) wild-type arrestin-3 (residues 1–408); or the indicated arrestin-3 mutants (residues 1–408). Lipofectamine2000 (Thermo Fisher) was used for transfection. After 48-hours, cells were incubated with phosphatase inhibitors (50 mM NaF and 10 mM Na_3_VO_4_) in PBS for 15 min at 37 °C and lysed with lysis buffer containing 50 mM Tris pH7.8, 2 mM EDTA, 250 mM NaCl, 10% glycerol, 0.5% NP-50, 20 mM NaF, 1 mM Na_3_VO_4_, 1 mM phenylmethanesulfonyl fluoride (PMSF) and 2 mM benzamidine. Sonication was used to further lyse the cells (60 Sonic Dismembrator, Fisher Scientific). The whole cell lysate were centrifuged at 10,000×*g* for 15 min and the supernatant was used for Western analysis. Activation of JNK3 was assessed using a phospho-JNK specific antibody (Cell Signaling #9251, 1:1000 dilution). The expression level of HA-ASK1, Flag-JNK3 and arrestin-3 were assessed by Western analysis using antibodies against the HA tag (Cell Signaling #C29F4, 1:1000 dilution), the Flag tag (Sigma #F3165, 1:500 dilution), arrestin (F4C1)^63^ or GFP (JL-8, Choltech #632381, 1:2000 dilution), respectively. The results were quantified using VersaDoc and QuantityOne software (Bio-Rad). The uncropped blots of one representative experiment are shown in Supplementary Fig. 8.

### DEER distance measurements

Cysteine substitutions of Ser13 and Ala392 were introduced into otherwise Cys-less arrestin-3 (residues 1–393). This variant was purified and spin-labeled with MTSL, as described previously^31^. Double electron electron resonance (DEER) spectroscopy data were collected using a Bruker E580 operating at Q-band and equipped with an EN5107D2 resonator. Samples contained 20% deuterated glycerol as a cryoprotectant and 100 µM protein with varying amounts of IP_6_ were run at 80 K following flash freezing in a dry ice and acetone mixture. Acquired raw dipolar evolution data were phase and background corrected, plotted and analyzed for distance distributions in the same way for each data set using the algorithms included in the LongDistances software program^64^ written by C. Altenbach (University of California-Los Angeles, CA). The upper reliable distance limit of 65 Å was determined based on the maximum data collection time (*t* = 4.5 µs) of the DEER experiments according to the equation *d* ≈ 5(t/2)1/3^65^ and is reflected in the *x*-axis of the distance distribution plot. The release of the C-tail causes the distances to spread out and possibly become longer than the observable range of our data.

### Microscale thermophoresis

Microscale thermophoresis (MST) was conducted using a NT.115 MST instrument (NanoTemper Technologies GmbH) equipped with green and blue filter sets. All arrestin-3 variants (residues 1–393; 20 µM in MST buffer (20 mM 3-morpholinopropane-1-sulfonic acid (MOPS) pH 7.5, 150 mM NaCl, and 1 mM TCEP) were labeled by adding 200 μL of 20 μM arrestin-3 to 1 μL of 40 mM Alexa Fluor C5 maleimide dye (final dye concentration, 200 µM; Molecular Probes, Eugene, OR) in DMSO and incubating the mixture at room temperature in the dark for 30 min. Free dye was separated from the protein-dye conjugate using a PD10 G25 column (GE Healthcare Bio-Sciences, Piscataway, NJ) equilibrated in MST buffer. Lauryl maltose neopentyl glycol (MNG-3; Anatrace, Maumee, OH) at 1% (w/v) in all solutions prevented the adherence of protein to the plasticware. The labeling stoichiometry was 0.95–2.0 dye molecules per arrestin-3 as estimated by spectrophotometry.

Titrations were usually accomplished by preparing 15 samples of IP_6_ in a 1:1 dilution scheme. A sixteenth sample with no IP_6_ established the baseline fluorescence or temperature jump. Labeled arrestin-3 (50 nM) and the reaction mixtures were equilibrated for 45 min then loaded into premium coated capillary tubes (NanoTemper). Data were acquired using 40% MST and 50% LED settings. A 5 s pre-IR phase was recorded, followed by a 60 sec phase with the IR laser on and a 5 s post-IR phase. In the case of the wild-type arrestin-3 construct, the pre-IR fluorescence varied strongly (~ 30% difference between the maximum and minimum intensity values) as a function of IP_6_ concentration, allowing this signal to be analyzed directly. The temperature-jump methodology was used to analyze data from the ΔN_IP6_ or T222C variants^66^. To prevent aggregation of the latter mutant, we included 0.5 mg/mL soybean trypsin inhibitor (Worthington Biochemical) in the MST buffer; this mutant also required an adjustment of the illumination protocol, featuring 75% LED power and an IR-on phase of only 30 s. Data were the average of at least two replicates. Data were analyzed in a version of PALMIST^66^ modified to include two-site binding models (manuscript submitted). MST figures were rendered using GUSSI^67^.

### Analytical ultracentrifugation

Purified arrestin-3 (residues 1–393) with or without IP_6_ (100 µM) was analyzed in an Optima XLI ultracentrifuge (Beckman Coulter, Brea, CA) equipped with a four-hole An-60 Ti rotor at 142,000×*g* at 4 °C. Samples were loaded into double-sector cells (path length of 1.2 cm) with charcoal-filled Epon centerpieces and sapphire windows. Sedfit (version 12.0) was used to analyze velocity scans using every scan from a total of between 250–300 scans^68^. Approximate size distributions were determined for a confidence level of *p* = 0.95, a resolution of *n* = 300, and sedimentation coefficients between 0.1 and 15 S. The frictional ratio was allowed to float.

### BRET measurements of arrestin-3 binding to receptors

Interactions between N-terminally Venus-tagged arrestin-3 (residues 1–408) and C-terminally RLuc8-tagged M2 muscarinic receptor or D2 dopamine receptor were determined by BRET, as described, using the highest (saturating) arrestin-3 concentrations^14^. Absolute levels of luminescence were used as the measure of the expression levels of RLuc-tagged receptors, whereas direct fluorescence was used to determine the expression of Venus-tagged arrestins, as described^14^. BRET was measured 15 min after the addition of 10 µM M2 agonist carbachol or 10 µM D2 agonist quinpirole, both of which were added along with 5 µM of the luciferase substrate coelenterazine-h. Luminescence and fluorescence were measured using Infinite F500 multimode plate reader (Tecan). Net BRET was calculated as the difference between BRET signal in the presence and absence of agonist to determine the expression of Venus-tagged arrestins, as described^14^. BRET was measured 15 min after the addition of 10 µM M2 agonist carbachol or 10 µM D2 agonist quinpirole, both of which were added in conjunction with 5 µM of the luciferase substrate coelenterazine-h. Luminescence and fluorescence were measured using Infinite F500 multimode plate reader (Tecan). Net BRET was calculated as the difference between BRET signal in the presence and absence of agonist.

### JNK3 binding of arrestin-3 variants

His tagged JNK3α2 (10 µg) was incubated with Ni-NTA resin for 2 h and wild-type or disulfide-containing arrestin-3 (10 µg) was added to the mixture with and without 100 µM IP_6_. The samples were washed with buffer (20 mM MOPS, pH 7.4, 150 mM NaCl, 25 mM imidazole) and eluted with 100 µl of the same buffer containing 250 mM imidazole. The eluate was methanol precipitated, and the JNK3 bound arrestin-3 (residues 1–393) was visualized by both Coomassie blue staining and Western blot, quantified using the QuantityOne software, and the data were analyzed using Prism. For the disulfide-linked bands, measurements were made in quadruplicate and one-way ANOVA was used to compare JNK3 binding to disulfide-containing arrestin-3. The uncropped blots of one representative experiment are shown in Supplementary Fig. 8.

### Data availability

The authors declare that all data supporting the findings of this study are available within the article and its Supplementary Information files. Coordinates and structure factors have been deposited in the Protein Data Bank with accession code 5TV1. Raw diffraction data are deposited with SBGrid with the accession code 10.15785/SBGRID/330. The additional data that support the findings of this study are available from the corresponding authors upon request.

## Electronic supplementary material

Supplementary Information

Description of Additional Supplementary Files

Supplementary Movie 1

Supplementary Movie 2
