# Supplementary material for: Structural basis of arrestin-3 activation and signaling
Source: Nat Commun. 2017 Nov 10;8:1427. doi: 10.1038/s41467-017-01218-8 (PMC5681653; doi:10.1038/s41467-017-01218-8)
Supplement: Supplementary file 1 — Supplementary Information [file 41467_2017_1218_MOESM1_ESM.pdf]

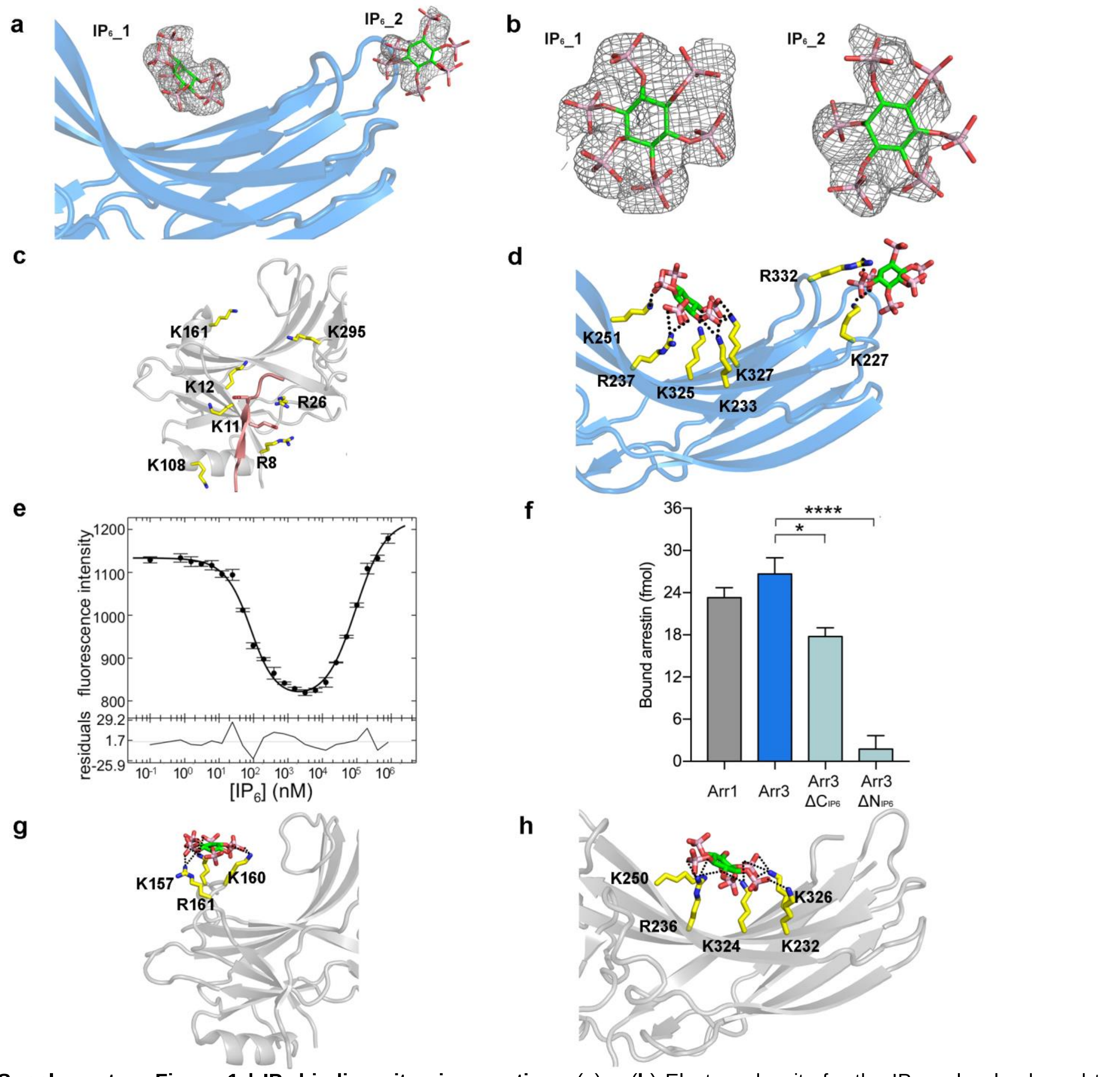

Supplementary Figure 1 | IP_6 binding sites in arrestins. (**a**) – (**b**) Electron density for the IP_6 molecules bound to arrestin-3. IP_6 molecules are superimposed on a $2F_o – F_c$ composite omit map calculated after refinement in Phenix and contoured at 1.2σ. (**c**) The arrestin-3 C-tail binds to the N-domain in basal state (PDB entry 3P2D[1]), contributing to the polar core and protecting the phosphate sensor. IP_6 or phosphopeptide binds at this same location, triggering the phosphate sensor and displacing the C-tail. (**d**) IP_6 binding sites in the arrestin-3 C-domain. (**e**) MST-measured fluorescence binding isotherm for IP_6 and wild-type arrestin-3. Circles in the upper panel represent the fluorescence-intensity average of three individual data acquisitions, error bars show S.D. The solid line is the fitted isotherm for the two-site model. The lower panel shows the residuals between the data and the fit. (**f**) Binding of the ΔN_{IP6} and ΔC_{IP6} arrestin-3 mutants to phosphorylated, active rhodopsin as compared to arrestin-1 and arrestin-3. The data were analyzed by one-way ANOVA. *, $p<0.05$; ****, $p<0.0001$. The ΔC_{IP6} variant binds to phosphorylated, activated rhodopsin at a level similar to wild-type arrestin-1, consistent with correctly-folded protein. The ΔN_{IP6} variant alters receptor-binding residues and binds rhodopsin only at low levels. Correct folding of the ΔN_{IP6} variant was validated by SEC of the purified protein. (**g**) IP_6 binding sites on arrestin-2 N-domain (PDB entry 1ZSH[2]) (**h**) IP_6 binding sites on arrestin-2 C-domain (PDB entry 1ZSH[2]).

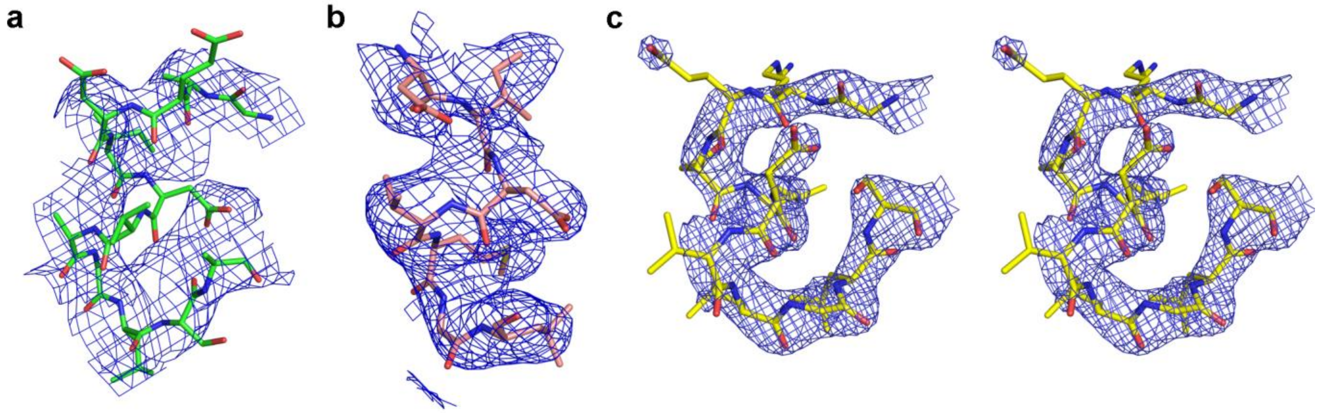

Supplementary Figure 2 | Electron density for the receptor-associated finger loop of arrestin as compared to the IP_6-bound trimer. Composite omit electron density is contoured at 1σ for (**a**) the finger loop of arrestin-1 in the structure of opsin-arrestin1 (PDB 4ZWJ[3]); (**b**) a peptide corresponding to the arrestin finger loop cocrystallized with opsin (PDB 4PXF[4]); (**c**) the finger loop of arresin-3 in the IP_6-activated trimer. Stereo images are presented in (c) for better visualization. The quality of the electron density in panels (**a**) – (**c**) differs significantly, likely reflecting different mobility of the finger loop in each structure determination. The quality of the electron density in panels (**a**) and (**b**) led to some controversy as to whether the finger loops was correctly assigned as a helix in these prior publications[3,4].

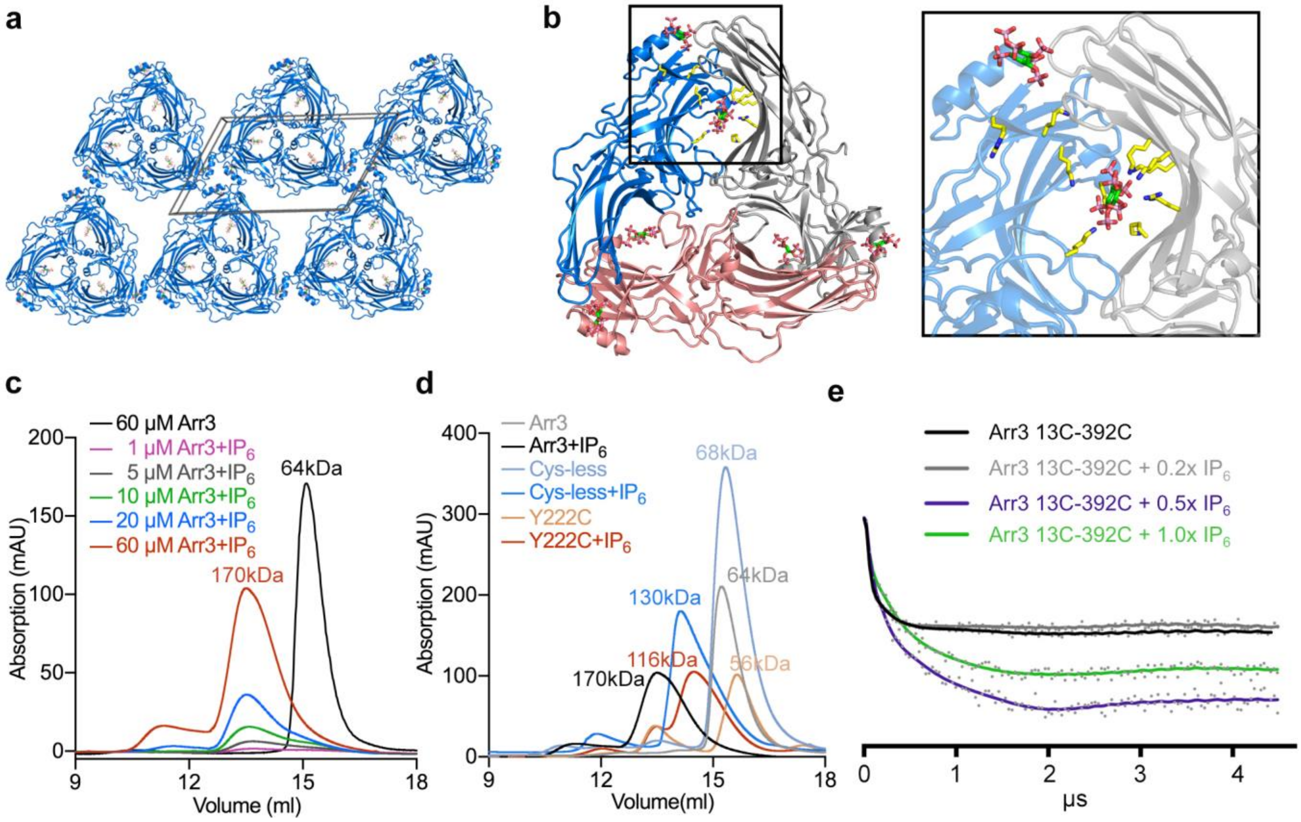

Supplementary Figure 3 | Evaluation of trimerization upon IP_6 binding to arrestin-3. (**a**) Packing of the arrestin-3 trimer into the crystal lattice. The unit cell is delineated with black lines. The crystal contacts are at non-functional regions of arrestin-3. Moreover, there is limited surface area involved in the crystal contacts. Together, this suggests that crystal packing does not influence trimer formation. (b) Arrestin-3 in the basal state (PDB entry 3P2D[1]) with the C tail (382-393) omitted is modeled as a trimer. The IP_6 binding interface is highlighted in the black rectangle. The N domain IP6 sites are not properly aligned for ideal IP6 binding. (**c**) SEC of wild-type arrestin-3 on a 24-mL Superdex S-200 increase column in buffer containing 100 μM IP_6 shows trimer formation in all arrestin-3 concentrations tested. The lowest concentration is 1μM arrestin-3, and is associated with a 2 mAu change in absorbance. (**d**) SEC of wild-type and Cys-less variants of arrestin-3 show that IP_6 addition to the Cys-less variants results in a loss of the high molecular weight peak that corresponds to the IP_6-dependent trimer. A shift is still observed, corresponding to a lower predicted molecular mass. (**e**) Background corrected dipolar evolution Q-band DEER data (dots) and the resulting fits (solid line) to the data using the LongDistances algorithms and software.

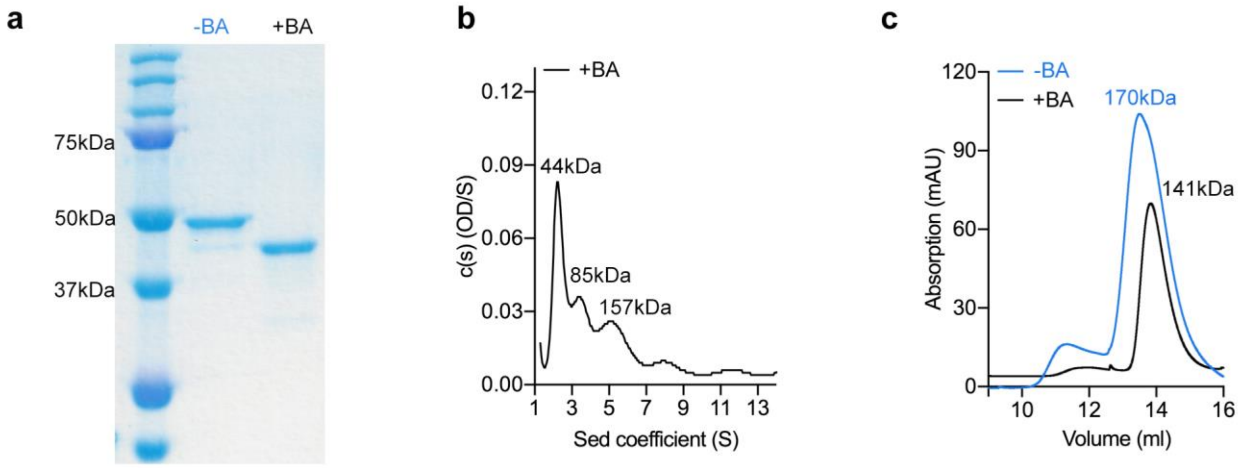

Supplementary Figure 4 | The impact of benzamidine on arrestin-3 oligomerization (**a**) SDS-PAGE analysis of arrestin-3 shows a time-dependent change in electrophoretic mobility in the presence of benzamidine. 60 µM arrestin-3 from the same protein preparation was split into two aliquots and incubated for 1 week at 4° C in buffer containing 20 mM MOPS pH 7.5, 150 mM NaCl, and 2 mM TCEP in the absence [5] or presence (+BA) of 1 mM benzamidine. The addition of benzamidine increased the mobility of arrestin-3 on the SDS-PAGE gel under both reducing (data not shown) and non-reducing conditions (shown). Mass spectrometry analysis of trypsin digested samples did not identify a rationale for this altered behavior (data not shown). (**b**) Benzamidine exposure of arrestin-3 correlates with a loss of trimerization and the formation of mixed (non-trimeric) oligomers. Representative SVAUC of benzamidine treated arrestin-3 shows sample heterogeneity potentially consistent with monomer-dimer-tetramer, in contrast to the trimer observed without benzamidine. The conversion between a trimeric form (no benzamidine) and dimer-tetramer (benzamidine) suggests that benzamidine promotes non-physiological oligomerization of arrestin-3 and does not stabilize the trimer. (**c**) The average molecular weight of benzamidine-treated arrestin-3 is a dimer. SEC of wild-type arrestin-3 in the presence of 100 µM IP_6 and 1 mM benzamidine (+BA) shows a smaller Stokes radius than arrestin-3 in the presence of 100 µM IP_6 but the absence of benzamidine. The elution volume of arrestin-3 in the presence of benzamidine is consistent with a dimer, while the elution volume of arrestin-3 in the absence of benzamidine is consistent with a trimer.

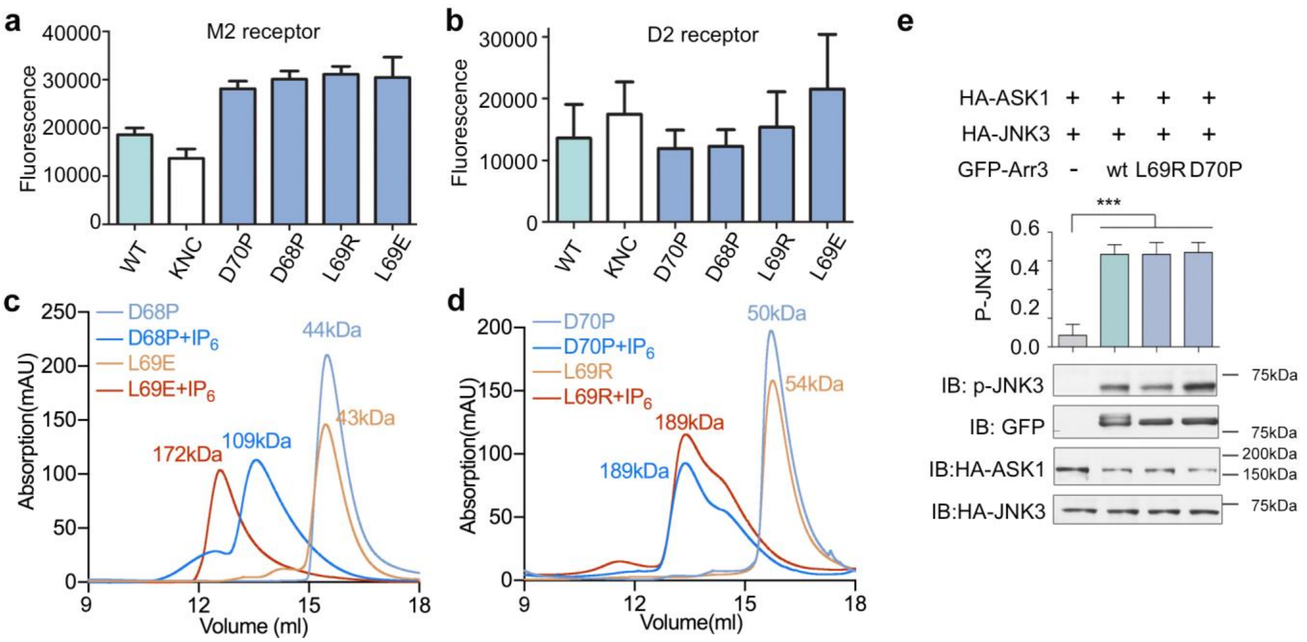

Supplementary Figure 5 | Role of the finger loop in arrestin-3 activation. (**a**) – (**b**) Expression of Venus-arrestin-3 variants in the BRET assays measured by fluorescence before agonist addition (λ_{ex}=500 nm, λ_{em}=535 nm) (n=3). (**c**) – (**d**) Size exclusion chromatograms of arrestin-3 finger loop variants in the presence and absence of IP_6 show that L69E exhibits an IP_6-dependent conversion to a high molecular weight species similar in elution volume to the wild-type trimer, L69R and D70P can convert to a high molecular weight species, but this conversion is incomplete, and D68P still shifts in molecular weight, but the shift is not consistent with the molecular weight of a trimer and more similar to a dimer. (**e**) Comparison of JNK3 activation by wild type arrestin-3 and the L69R and D70P finger loop mutants. JNK3 activation was assessed by measuring the phospho-JNK3 (pp-JNK3) level in COS7 cells co-transfected with HA-ASK1, HA-JNK3 and either GFP, Venus-tagged wild type arrestin-3, or the Venus-tagged arrestin-3 loop mutants. The data are from four independent experiments. The JNK3 phosphorylation was compared using one-way ANOVA followed by Bonferroni post hoc test with correction for multiple comparison. ***, $p<0.001$ to GFP. There were no significant differences between either of the mutants and wild-type arrestin-3.

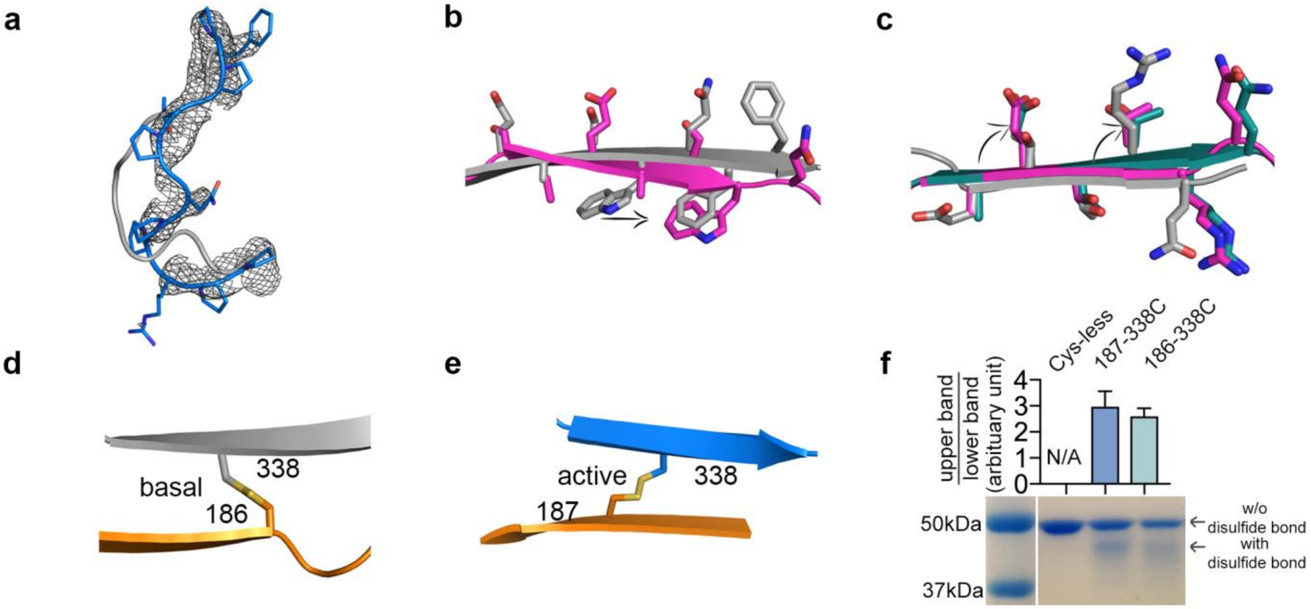

Supplementary Figure 6 | Arrestin switch regions. (**a**) Conformational changes in aSwI. Composite omit electron density for IP_6-activated arrestin-3 contoured at 0.8σ shown superimposed on the model of aSwI from IP_6-activated arrestin-3 (*blue*) and basal arrestin-3 (*grey,* PDB entry 3P2D[1]) (**b**) Comparison of aSwIIb of arrestin-1 in the receptor-activated (*magenta*, PDB entry 4ZWJ[3]) and basal (PDB entry 1CF1[6]) states of the protein. (**c**) Comparison of aSwIIb of arrestin-2 in the phosphopeptide-activated state (*magenta*, PDB entry 4JQI[7]), the basal state with the aSwIIb in the active conformation (*green*, PDB entry 1JSY[8]) and the basal state with the aSwIIb in the basal conformation (*grey*, PDB entry 1G4M[9]). (**d**) – (**e**) The introduction of a cysteine at position 338 in conjunction with cysteine substitution at position 186 or 187 is predicted to promote the formation of disulfides that stabilize aSwIIb in the (**d**) basal (Cys 186) or (**e**) active (Cys 187) conformation, respectively. (**f**) Comparison of non-reducing SDS-PAGE of the disulfide-trapped arrestin-3 variants reveals heterogeneous migration, consistent with the presence of the disulfide bond. Importantly, the ratio between the population without disulfide bond (upper band) versus the population with disulfide bond (lower band) is comparable between the 187-338C and 186-338C mutants.

a

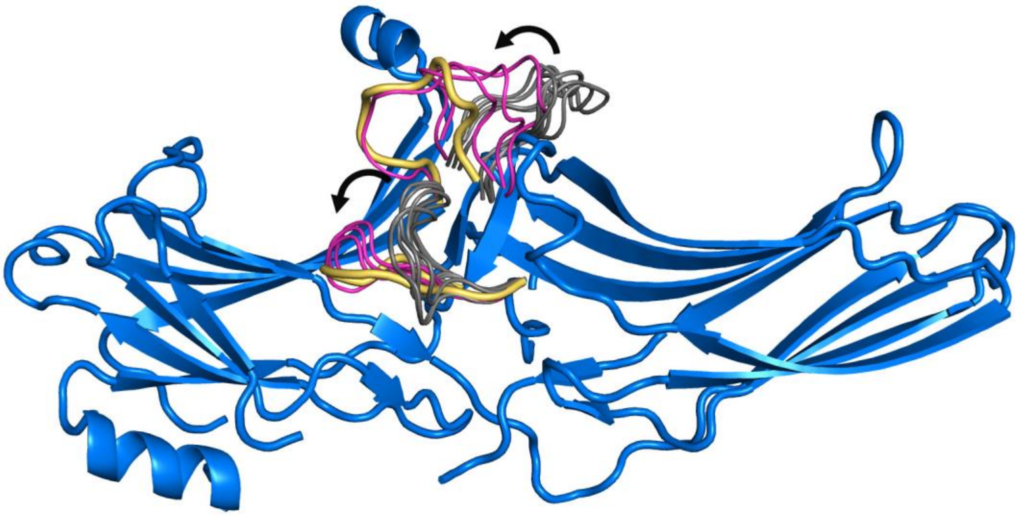

Supplementary Figure 7 | The position of middle loop and lariat loop in published arrestin structures. (**a**) Overlay of middle loop and lariat loop of all published arrestin structures. *Grey*: PDB entries 1CF1[6], 1JSY[8], 1ZSH[2], 3P2D[1], 1G4M[9], 3UGX[10]; *Magenta:* PDB entries 4ZRG[11], 4JQI[7], 4J2Q[12], 4ZWJ[3]; *Yellow*: IP_6 activated arrestin-3.

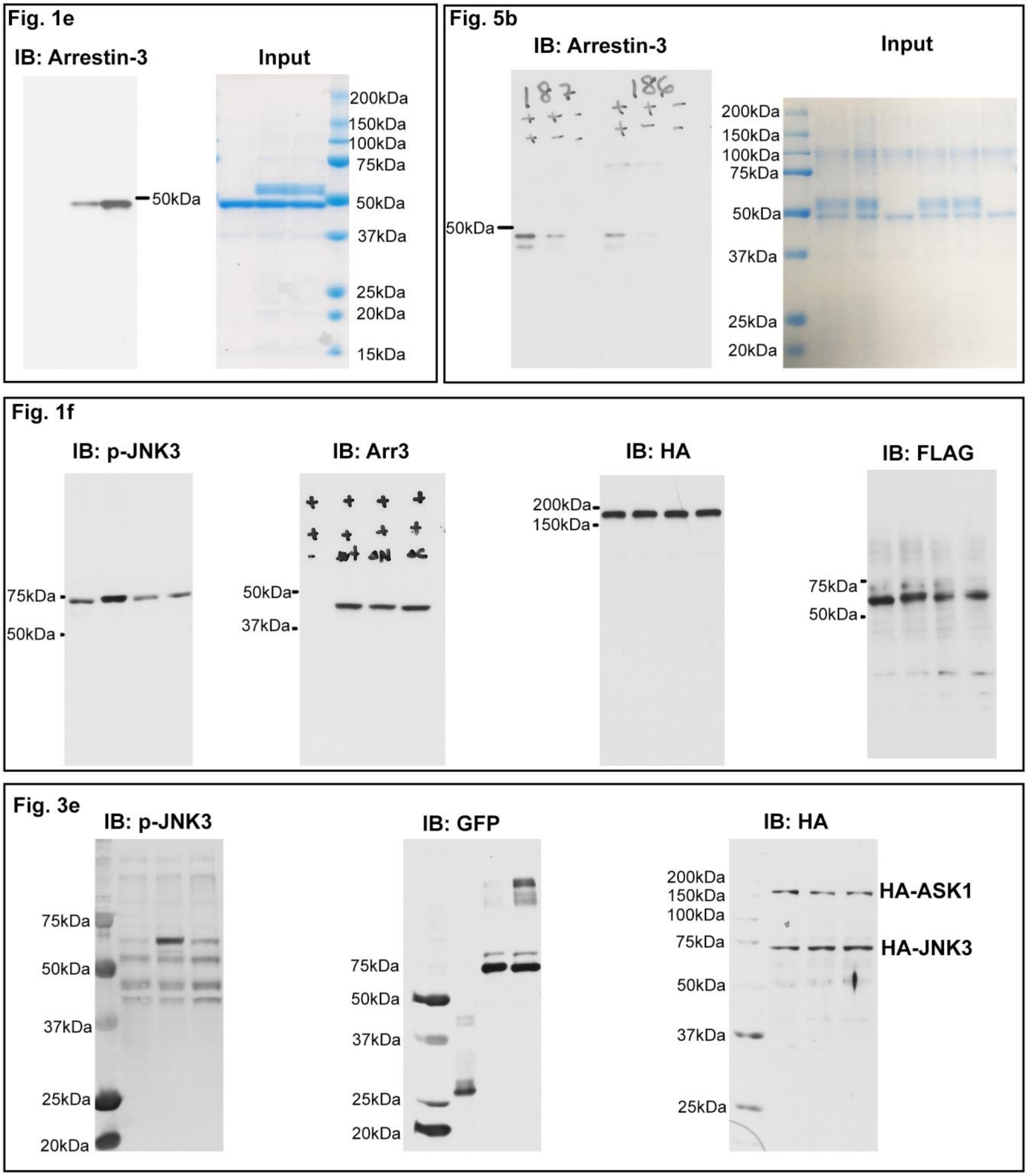

Supplementary Figure 8 | Full blots and gels of data presented in main text. The full blots and gels in Fig. 1e, 1f, 5b, 3e are displayed here where portions are presented in the maintext.

Supplementary Table 1.

Protein	Globular weight (no IP_6)	Oligomer	Globular weight (+ IP_6)	Oligomer
wild-type (60 µM)	64.0 ± 5.9 kDa	monomer	169.6 ± 9.7 kDa	trimer
wild-type + BA			141.1 ± 9.0 kDa	dimer
ΔN_{IP6}	57.4 ± 2.7 kDa	monomer	66.5 ± 3.7 kDa	monomer
ΔC_{IP6}	59.2 ± 3.9 kDa	monomer	54.6 ± 3.0 kDa	monomer
Cys-less	67.5 ± 4.4 kDa	monomer	130.0 ± 2.5 kDa	dimer
Cys-less + T222C	56.4 ± 4.0 kDa	monomer	115.7 ± 14.9 kDa	dimer
D68P	43.5 ± 3.9 kDa	monomer	109.0 ± 1.2 kDa	dimer
L69R	53.5 ± 1.6 kDa	monomer	189.0 ± 4.7 kDa	trimer
L69E	42.6 ± 1.5 kDa	monomer	172.2 ± 6.2 kDa	trimer
D70P	49.6 ± 5.1 kDa	monomer	189.0 ± 3.7 kDa	trimer

Supplementary Table 1 | Average molecular weights of wild-type and mutant arrestin in the absence and presence of IP_6 as measured by SEC. Arrestin-3 migrated at a higher apparent molecular weight as compared to standards, a feature shared with its migration on SDS-PAGE. The most likely oligomerization state under each condition is indicated.

Supplementary Table 2.

Protein structure	Phosphate sensor	Activation sensor
P44 arrestin	activated by C-tail truncation, which reduces the contacts that stabilize the basal conformation	not activated
arrestin-2 + V_2R peptide	activated by interaction with peptide-attached phosphates (native activator)	not activated
arrestin-1 + rhodopsin	activated by mutations in the C-tail	activated by interaction with receptor (native activator)
arrestin-3 + IP_6	activated by interaction with IP_6 phosphates (native activator)	activated by interaction with sister protomers in the trimer (native activator)

Supplementary Table 2 | Mechanisms of sensor activation and interdomain rotation in arrestin crystal structures. How each sensor was activated is indicated.

Supplementary Table 3.

**Finger loop mutation**	
L69Rf	CTATGGCCGCGAAGACCGGGACGTGCTGGGCTTG
L69Ef	CTATGGCCGCGAAGACGAGGACGTGCTGGGCTTG
D68Pf	CGCTATGGCCGCGAACCCCTGGACGTGCTGGGC
D70Pf	GGCCGCGAAGACCTGCCCGTGCTGGGCTTGTCC
**N domain IP_6 binding mutation**	
R8Qf	ACCACCATGGGGGAGAAACCCGGGACCCAGGTCTTCGCGGCATCGAGT
K108Qr	GGCATGCTGGCCCAGCTGCCTCAGCAGCCGCTC
K161Qr	GACGTACGGAGTTCCGTTGGTGGCTTTTCTCTTCTAG
K295Qf	GCTCTAGATGGGAAGCTCCAGCACGAGGACACCAAC
**C domain IP_6 binding mutation**	
K233Qf, R237Qf	CCAAGACTGTCAAGAAGATCCAAGTCTCTGTGCAACAGTACGCCGACAT CTGCCTCTTC
K251Qr	CTGAGCCACGGGGCACTGGTACTGGGCGGTGCTGAAGAGGCAGATGTC
K325Qf, K327Qf	GGTGTCCTACAGGGTCCAGGTGCAGCTGGTGGTGTCACGAGGC
**Disulfide trapping mutantation**	
A185Cr	CATGAGGAAGTGGCGTGTGGTTTCACATGAAGGCTGGGGCCC
E186Cr	CATGAGGAAGTGGCGTGTGGTACAAGCTGAAGGCTGGGGCCC
T187Cr	CATGAGGAAGTGGCGTGTGCATTCAGCTGAAGGCTGGGGCCC
V338Cr	GATGTGATCATGGGGCTTGGGGTGCATGAGAACAAATGGCAGCTCACATGAGAC ATCCCCGCCT

Supplementary Table 3 | The sequence of primers used to introduce mutations in arrestin-3.

Supplementary methods:

Rhodopsin binding of radiolabelled arrestin-3. A detailed description of in vitro transcription, translation, preparation of phosphorylated rhodopsin and binding assays has been reported[13]. Briefly, 1 nM arrestin-1 or arrestin-3 variant (50 fmol) was incubated with 0.3 mg of phosphorylated light-activated rhodopsin in 50 µl of 50 mM Tris-HCL, pH 7.4, 100 mM potassium acetate, 1 mM EDTA, 1 mM DTT for 5 min at 37 °C under room light. Samples were cooled on ice. Bound and free arrestin were separated at 4 °C by size exclusion chromatography on 2 ml Sepharose 2B-CL column. Arrestin eluting with the rhodopsin-containing membranes was quantified by liquid scintillation counting. Non-specific binding was determined in samples where rhodopsin was omitted and subtracted from the total binding.

Supplementary references:

1 Zhan, X., Gimenez, L. E., Gurevich, V. V. & Spiller, B. W. Crystal structure of arrestin-3 reveals the basis of the difference in receptor binding between two non-visual subtypes. *J Mol Biol* **406**, 467-478(2011).

2 Milano, S. K., Kim, Y. M., Stefano, F. P., Benovic, J. L. & Brenner, C. Nonvisual arrestin oligomerization and cellular localization are regulated by inositol hexakisphosphate binding. *J Biol Chem* **281**, 9812-9823(2006).

3 Kang, Y. *et al.* Crystal structure of rhodopsin bound to arrestin determined by femtosecond X-ray laser. *Nature* **523**, 561-567(2015).

4 Szczepek, M. *et al.* Crystal structure of a common GPCR-binding interface for G protein and arrestin. *Nat Commun* **5**, 4801(2014).

5 Stowell, S. R. *et al.* Ligand reduces galectin-1 sensitivity to oxidative inactivation by enhancing dimer formation. *J Biol Chem* **284**, 4989-4999(2009).

6 Hirsch, J. A., Schubert, C., Gurevich, V. V. & Sigler, P. B. The 2.8 A crystal structure of visual arrestin: a model for arrestin's regulation. *Cell* **97**, 257-269(1999).

7 Shukla, A. K. *et al.* Structure of active beta-arrestin-1 bound to a G-protein-coupled receptor phosphopeptide. *Nature* **497**, 137-141(2013).

8 Milano, S. K., Pace, H. C., Kim, Y. M., Brenner, C. & Benovic, J. L. Scaffolding functions of arrestin-2 revealed by crystal structure and mutagenesis. *Biochemistry* **41**, 3321-3328(2002).

9 Han, M., Gurevich, V. V., Vishnivetskiy, S. A., Sigler, P. B. & Schubert, C. Crystal structure of beta-arrestin at 1.9 A: possible mechanism of receptor binding and membrane Translocation. *Strucutre* **9**, 869-880(2001).

10 Granzin, J. *et al.* Crystal structure of p44, a constitutively active splice variant of visual arrestin. *J Mol Biol* **416**, 611-618(2012).

11 Granzin, J., Stadler, A., Cousin, A., Schlesinger, R. & Batra-Safferling, R. Structural evidence for the role of polar core residue Arg175 in arrestin activation. *Sci Rep* **5**, 15808(2015).

12 Kim, Y. J. *et al.* Crystal structure of pre-activated arrestin p44. *Nature* **497**, 142-146(2013).

13 Vishnivetskiy, S. A. *et al.* How does arrestin respond to the phosphorylated state of rhodopsin? *J Biol Chem* **274**, 11451-11454(1999).
