# Supplementary material for: Structural basis of arrestin-3 activation and signaling
Source: Nat Commun. 2017 Nov 10;8:1427. doi: 10.1038/s41467-017-01218-8 (PMC5681653; doi:10.1038/s41467-017-01218-8)
Supplement: Supplementary file 2 — Description of Additional Supplementary Files [file 41467_2017_1218_MOESM2_ESM.pdf]

Description of Additional Supplementary Files

File Name: Supplementary Movie 1
Description: A morph between basal arrestin and active arrestin highlights how different conformations are available to effectors. The N-domain is in blue, the C-domain is in grey, the receptor-binding ridge and finger loop is in green, arrestin switch regions are in orange, and the register-shifted β-strand is in magenta.

File Name: Supplementary Movie 2
Description: A morph between basal and active arrestin after a 180° rotation around the y-axis.
